# Photoinduced hole transfer from tris(bipyridine)ruthenium dye to a high-valent iron-based water oxidation catalyst†
†Electronic supplementary information (ESI) available. See DOI: 10.1039/c8fd00167g


**DOI:** 10.1039/c8fd00167g

**Published:** 2019-04-05

**Authors:** Sergii I. Shylin, Mariia V. Pavliuk, Luca D’Amario, Igor O. Fritsky, Gustav Berggren

**Affiliations:** a Department of Chemistry – Ångström Laboratory , Uppsala University , P. O. Box 523 , 75120 Uppsala , Sweden . Email: sergii.shylin@kemi.uu.se ; Email: gustav.berggren@kemi.uu.se; b Physics Department , Free University Berlin , Arnimallee 14 , 14195 Berlin , Germany; c Department of Chemistry , Taras Shevchenko National University of Kyiv , Volodymyrska 64 , 01601 Kiev , Ukraine . Email: ifritsky@univ.kiev.ua; d PBMR Labs Ukraine , Murmanska 1 , 02094 Kiev , Ukraine

## Abstract

An efficient water oxidation system is a prerequisite for developing solar energy conversion devices. Using advanced time-resolved spectroscopy, we study the initial catalytic relevant electron transfer events in the light-driven water oxidation system utilizing [Ru(bpy)_3_]^2+^ (bpy = 2,2′-bipyridine) as a light harvester, persulfate as a sacrificial electron acceptor, and a high-valent iron clathrochelate complex as a catalyst. Upon irradiation by visible light, the excited state of the ruthenium dye is quenched by persulfate to afford a [Ru(bpy)_3_]^3+^/SO_4_˙^–^ pair, showing a cage escape yield up to 75%. This is followed by the subsequent fast hole transfer from [Ru(bpy)_3_]^3+^ to the Fe^IV^ catalyst to give the long-lived Fe^V^ intermediate in aqueous solution. In the presence of excess photosensitizer, this process exhibits pseudo-first order kinetics with respect to the catalyst with a rate constant of 3.2(1) × 10^10^ s^–1^. Consequently, efficient hole scavenging activity of the high-valent iron complex is proposed to explain its high catalytic performance for water oxidation.

## Abstract 2

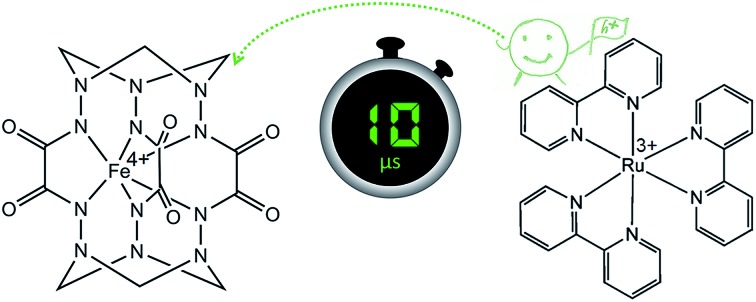
Fast visible light-driven water oxidation catalyzed by the Fe^IV^ cage complex relies on its efficient hole scavenging activity in the system utilizing [Ru(bpy)_3_]^2+^ as a photosensitizer.

## Introduction

Climate change, driven by the greenhouse effect and increasing global energy consumption, has become the greatest challenge humanity has ever faced. Most analysts believe that the world’s demand for energy will keep growing, providing a strong motivation for research into the development of carbon-neutral and carbon-negative energy sources.^1^ In this regard, artificial photosynthesis, harnessing sunlight and water as practically infinite resources, is becoming an attractive way to achieve the sustainable energy goals.^2^–^4^ To date, the development of efficient water oxidation catalysts remains a challenging task as oxidation of water to dioxygen is a complicated process involving four electron transfer steps coupled with the release of four protons.^5^ The classic homogenous photocatalytic water oxidation system consists of a water oxidation catalyst (WOC), a photosensitizer and a sacrificial electron acceptor. Thus, in addition to the challenges associated with bond making and breaking during catalysis, sustainable generation of a long-lived charge-separated state in aqueous solution is required to perform the desired catalytic process.^6^,^7^ Moreover, the photosensitizer must be able to oxidize the WOC, having a redox potential *E*_1/2_ > 1.23 V *versus* the normal hydrogen electrode (NHE). These requirements can be met by ruthenium polypyridine complexes, and consequently they have been widely employed as efficient visible light-driven photosensitizers in conjunction with various WOCs.^8^–^10^ Among these complexes, tris(bipyridine)ruthenium ([Ru(bpy)_3_]^2+^) is the most common light harvester, with a relatively high oxidation potential *E*_1/2_ = 1.26 V *versus* NHE for the [Ru(bpy)_3_]^3+^/[Ru(bpy)_3_]^2+^ redox couple.^11^ Due to the long lifetime *τ* ∼ 10^–6^ s of its metal-to-ligand charge transfer (MLCT) excited state, [Ru(bpy)_3_]^2+^ may be easily converted to [Ru(bpy)_3_]^3+^ under illumination, by electron acceptors such as persulfate.^12^ However, there is still room for improving the ability of the WOC to efficiently re-reduce [Ru(bpy)_3_]^3+^ to [Ru(bpy)_3_]^2+^ and perform sustained water oxidation.

**Fig. 1:**
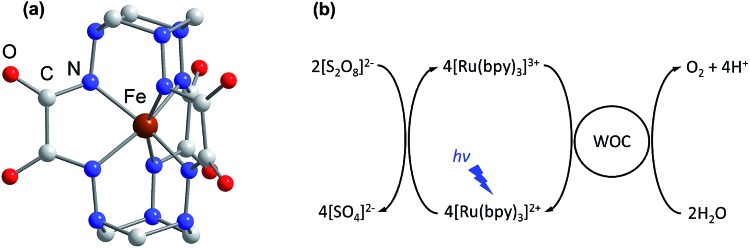
(a) The molecular structure of the complex anion [Fe^IV^(L–6H)]^2–^ reported previously.^35^ H atoms are omitted for clarity. (b) The photocatalytic cycle of water oxidation to dioxygen by persulfate with [Ru(bpy)_3_]^2+^ as the photosensitizer and WOC.

Over the past few decades various heterogeneous WOCs, generally considered more suitable for practical use, and homogeneous analogues, often considered more suitable for mechanistic studies, have been designed. Transition-metal oxides of Groups 7, 8, and 9 were observed early on to have good catalytic properties for oxygen evolution.^13^ Among these metals, a privileged position belongs to manganese,^14^ as it constitutes the cofactor of photosystem II in living organisms, as well as ruthenium and iridium,^15^,^16^ located diagonally in the periodic table relative to manganese. Oxides of these two noble metals have found their application in commercial proton-exchange membrane electrolyzers due to their stability over a wide pH range, despite their high price and harm to the environment.^17^ Like their heterogeneous counterparts, the best-characterized homogeneous WOCs are based on ruthenium and iridium.^18^,^19^ Due to the high costs and low abundance of these metals, there is considerable interest in the use of cheap base metal complexes for water oxidation. To date, a handful of cobalt,^20^ manganese,^21^,^22^ copper,^23^ nickel^24^ and iron compounds have been studied with respect to their potential as WOCs. However, iron, being the most abundant and cheap transition metal, is the least used in molecular WOCs, arguably due to the low stability of iron complexes under oxidative conditions. Only a few molecular iron compounds have been documented as catalysts for chemical,^25^–^29^ electrochemical^30^–^32^ and photochemical water oxidation.^33^,^34^ The most noteworthy iron-based WOCs are TAML (tetraamido macrocyclic ligand) complexes, where enhanced catalyst stability is achieved by utilizing a tetradentate macrocycle, and high-valent iron catalytic intermediates are supported by deprotonated N donor atoms.^25^,^32^,^33^ We recently reported fast light-driven water oxidation catalyzed by the exceptionally stable iron(iv) cage complex [Fe^IV^(L–6H)]^2–^, whose structure is depicted in Fig. 1a.^35^ In the tris(bipyridine)ruthenium dye photo-oxidant system (Fig. 1b), it shows catalytic performance with a turnover frequency (TOF) of 2.27 s^–1^ and a maximum turnover number (TON) of 365.^36^ Its high efficiency has been attributed to the robust clathrochelate ligand that prevents the catalyst from rapid degradation, as has been shown by UV-vis, EPR, Mössbauer spectroscopy, ESI mass spectrometry and DLS studies. The relatively high rate of water oxidation by the catalyst is attributable to its mild water oxidation overpotential of 0.39 V, compared with other iron-based WOCs. In this contribution, we report the hole scavenging activity of the iron(iv) catalyst in the ruthenium dye water oxidation system in order to shed further light on its catalytic efficiency.

## Experimental

### Synthesis

The general synthesis pathway of iron clathrochelates bearing the [Fe^IV^(L–6H)]^2–^ complex anion has been described previously.^35^ In short, the compound Na_2_[Fe^IV^(L–6H)]·2H_2_O used in the present work as the WOC, was synthesized as follows. Fe(ClO_4_)_3_·H_2_O (0.372 g, 1 mmol, dissolved in 5 ml of water) was added to a warm solution of oxalodihydrazide (0.354 g, 3 mmol, dissolved in 15  ml of water). Then, a solution of NaOH (0.2 g, 5 mmol in 5 ml of water) and an aqueous formaldehyde solution (37%, 0.67 ml, 9 mmol) were added to the resulting mixture. The reaction mixture was stirred for 2 h at room temperature, and then filtered off and the filtrate was removed on a rotary evaporator. The resulting residue was washed with chloroform and ethanol, air dried, and recrystallized from water. The composition of the complex was confirmed by comparing its IR spectrum with that reported previously, and elemental analysis.

### Oxygen evolution

Oxygen evolution was detected polarographically using a standard Clark-type oxygraph electrode (Hansatech Instruments) placed in a thermostated cell and separated from the sample solution by an oxygen-permeable Teflon membrane. The signal was recorded every 0.1 s using the Oxygraph software package. An air-saturated aqueous solution was used for calibration of the electrode. All of the experiments were carried out at 20 °C. The cell was purged with argon gas before each experiment, and the solution in the cell (1 ml) was continuously stirred. During light-driven oxygen detection experiments, the buffered solution (borate buffer, 0.1 M, pH 8.0) in the working cell contained [Ru(bpy)_3_](ClO_4_)_2_, Na_2_S_2_O_8_ and Na_2_[Fe^IV^(L–6H)], the concentrations of which were varied as described in the next section. Visible light LEDs (*λ* = 450(10) nm, 3 W) were used as illumination sources in the photoinduced reactions. For Ru^III^-induced oxygen evolution, an aqueous solution of Na_2_[Fe^IV^(L–6H)] was added to the freshly prepared solutions of [Ru(bpy)_3_](ClO_4_)_3_ (1 mM) in borate buffer (0.1 M, pH 8.0).

### Steady-state and time-resolved UV-vis spectroscopy

Steady-state UV-vis spectra were recorded using a Varian Cary 50 spectrometer in a 1 mm cuvette. Time-resolved experiments were carried out using a nanosecond laser spectroscopy setup. The solutions under study were measured in a 1 mm quartz cuvette using a pump-probe methodology. Microsecond transient absorption kinetics were recorded by a Q-switched Nd:YAG laser (Quanta Ray Pro-230), which produced tripled frequency pulses with *λ* = 355 nm (13 ns). The laser was coupled with an optical parametric oscillator to obtain the desired wavelength (460 nm) for the pump light. An excitation light power of 30 mJ per pulse was used in all of the experiments. The data were collected using an Edinburgh Instruments LP900 spectrometer equipped with a 450 W Xe lamp used as a probe light source. Light was collected using an Andor CCD camera for TA spectra and a Hamamatsu R928 photomultiplier tube for kinetic traces. Kinetic traces of the optical density at 420 nm (shoulder of the absorption band of [Ru(bpy)_3_]^2+^) were studied for solutions containing [Ru(bpy)_3_](ClO_4_)_2_ (0.04 mM), Na_2_S_2_O_8_ (0.4 mM) and Na_2_[Fe^IV^(L–6H)] (1.0–3.0 μM). For [Ru(bpy)_3_]^2+^ luminescence studies, the transient emission spectra and corresponding kinetic traces at 650 nm were recorded (a 580 nm long pass filter was used to block the scattering of pump light).

### Dynamic light scattering

DLS experiments were performed using a Zetasizer Nano S scattering system (Malvern Instruments Ltd) that used a uni-phase He–Ne laser (633 nm; 4 mW) working in cross auto-correlation mode. The scattering angle was set to 90° with respect to the incident laser. The intensity correlation curves were analyzed with the Zetasizer family software. The size measurement range was from 0.3 nm to 10 μm.

## Results

### UV-vis spectroscopy studies

**Fig. 2:**
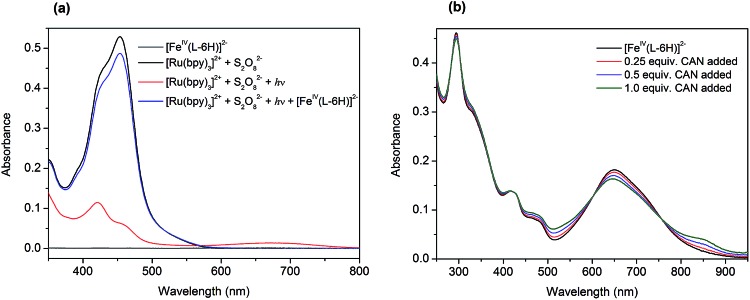
(a) UV-vis spectra demonstrating the photochemical oxidation of [Ru(bpy)_3_]^2+^ (0.05 mM) to [Ru(bpy)_3_]^3+^ by persulfate (0.2 mM) with the following reduction by the catalyst (0.5 μM). The spectrum of the catalyst (0.5 μM) is given for comparison in grey. (b) UV-vis spectra demonstrating titration of the catalyst [Fe^IV^(L–6H)]^2–^ (0.02 mM) by CAN.

At the outset, we performed a spectroscopy assay of the tris(bipyridine)ruthenium dye photo-oxidant system without the Fe^IV^ catalyst. The UV-vis spectrum of the aqueous solution containing [Ru(bpy)_3_](ClO_4_)_2_ (0.05 mM) and Na_2_S_2_O_8_ (0.2 mM) recorded in darkness is shown in Fig. 2a (black). It exhibits two overlapping MLCT bands at 420 nm and 455 nm, characteristic of [Ru(bpy)_3_]^2+^. Illumination of this assay solution using blue light LEDs (*λ* ∼ 450 nm) resulted in a rapid loss of color, and in a few seconds nearly 90% of the [Ru(bpy)_3_]^2+^ converted to [Ru(bpy)_3_]^3+^ (Fig. 2a, red):

1 [Ru(bpy)_3_]^2+^ + *hν* → [Ru(bpy)_3_]^2+^*

2 [Ru(bpy)_3_]^2+^* + S_2_O_8_^2–^ → [Ru(bpy)_3_]^3+^ + SO_4_^2–^ + SO_4_˙^–^

3 [Ru(bpy)_3_]^2+^ + SO_4_˙^–^ → [Ru(bpy)_3_]^3+^ + SO_4_^2–^

The photochemically generated [Ru(bpy)_3_]^3+^ is relatively stable in water, as can be concluded from the follow-up spectroscopic observation. Subsequent addition of 0.01 equivalents of Na_2_[Fe^IV^(L–6H)] (0.5 μM) to this solution resulted in immediate restoration of the yellow color, as practically all of the [Ru(bpy)_3_]^3+^ was re-reduced to [Ru(bpy)_3_]^2+^ (Fig. 2a, blue). We proposed previously that the catalyst [Fe^IV^(L–6H)]^2–^ is oxidized by [Ru(bpy)_3_]^3+^ to the intermediate state [Fe^V^(L–6H)]^–^, as a first step in the catalytic process.^36^ Alternatively, [Fe^IV^(L–6H)]^2–^ can be gently oxidized by (NH_4_)_2_[Ce(NO_3_)_6_] (CAN), in which case it is possible to monitor the Fe based redox process by UV-vis spectroscopy as CAN does not have absorption bands overlapping with the bands characteristic of [Fe^IV^(L–6H)]^2–^. After the addition of 0.25–1 equivalents of CAN to [Fe^IV^(L–6H)]^2–^ in nitric acid (pH 1.5), an increase in absorption is observed around 550 nm and 850 nm at the same time as the intensity of the [Fe^IV^(L–6H)]^2–^ band at 650 nm decreases (Fig. 2b). The newly formed absorption bands can be assigned to [Fe^V^(L–6H)]^–^, as has been shown previously using EPR and Mössbauer spectroscopy in a Ru^III^-induced oxidation assay.^36^

### Photochemical and chemical water oxidation

Light-induced water oxidation was demonstrated in an aqueous solution containing [Fe^IV^(L–6H)]^2–^, [Ru(bpy)_3_]^2+^, and S_2_O_8_^2–^ in borate buffer (pH 8.0) in a cell equipped with a Clark electrode.^36^ In this follow-up study, we have optimized the conditions for photochemical water oxidation by varying the concentrations of photosensitizer and sacrificial electron acceptor. The TOF values reach saturation at 2.35 s^–1^, at concentrations of [Ru(bpy)_3_]^2+^ above 0.3 mM and concentrations of S_2_O_8_^2–^ above 2 mM (Fig. S1 in the ESI†). The evolution of dioxygen during the photocatalytic water oxidation using [Fe^IV^(L–6H)]^2–^ reaches a plateau after 150–240 s, as the catalytic system becomes inactive after >300 turnovers. Our dynamic light scattering (DLS) studies do not show any traces of nanoparticles after oxygen evolution has stopped (Fig. S2 in the ESI†), it thus appears that the [Fe^IV^(L–6H)]^2–^ catalyst degrades to other soluble iron complex(-es) unable to perform water oxidation. Still, since it is known that iron hydroxide is catalytically active,^37^ we performed control photochemical water oxidation experiments under nearly identical conditions, but using FeCl_3_ instead of [Fe^IV^(L–6H)]^2–^ as a precatalyst giving hematite at pH 8.0. When 1 μM FeCl_3_ is used, oxygen evolution is indeed observed, but the TON and TOF are almost 80% lower than those observed for [Fe^IV^(L–6H)]^2–^ (Fig. S3†).

**Fig. 3:**
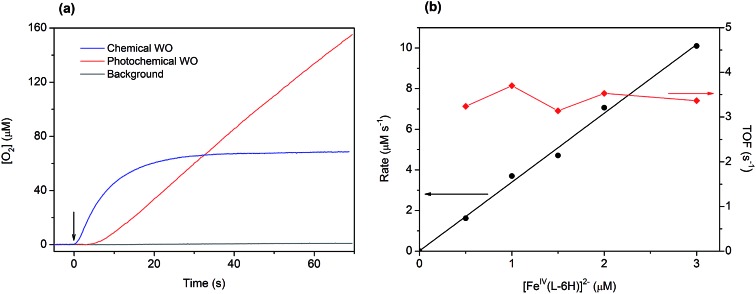
(a) Traces of oxygen evolution obtained in photochemical (red trace: [Fe^IV^(L–6H)]^2–^ (1 μM), [Ru(bpy)_3_]^2+^ (0.2 mM) and S_2_O_8_^2–^ (2 mM)) and chemical water oxidation experiments (blue trace: [Fe^IV^(L–6H)]^2–^ (1.5 μM) and [Ru(bpy)_3_]^3+^ (1 mM)). Oxygen evolution in the absence of catalyst is given for comparison (grey trace: [Ru(bpy)_3_]^2+^ (0.2 mM) and S_2_O_8_^2–^ (2 mM)). The arrow indicates the beginning of the reaction (the start of illumination or addition of the oxidant, respectively). (b) Initial water oxidation rates (circles) and TOF (diamonds) as a function of the catalyst concentration for chemical water oxidation using [Ru(bpy)_3_]^3+^ (1 mM).

The kinetics of water oxidation using the catalyst [Fe^IV^(L–6H)]^2–^ has been evaluated using the one-electron oxidant [Ru(bpy)_3_](ClO_4_)_3_. Addition of [Fe^IV^(L–6H)]^2–^ (0.2–5 μM) to a solution of [Ru(bpy)_3_]^3+^ (1 mM) at pH 8.0 leads to the immediate formation of oxygen that can be detected using the Clark electrode (Fig. 3a). At a catalyst concentration of 1.5 μM, the maximum TON of 45 was reached. As in the photochemical water oxidation studies,^36^ the initial rates of water oxidation by [Ru(bpy)_3_]^3+^ exhibit a linear dependence on the catalyst concentration (Fig. 3b) with a first-order rate constant of 3.3(1) s^–1^.

### Transient absorption spectroscopy studies

**Fig. 4:**
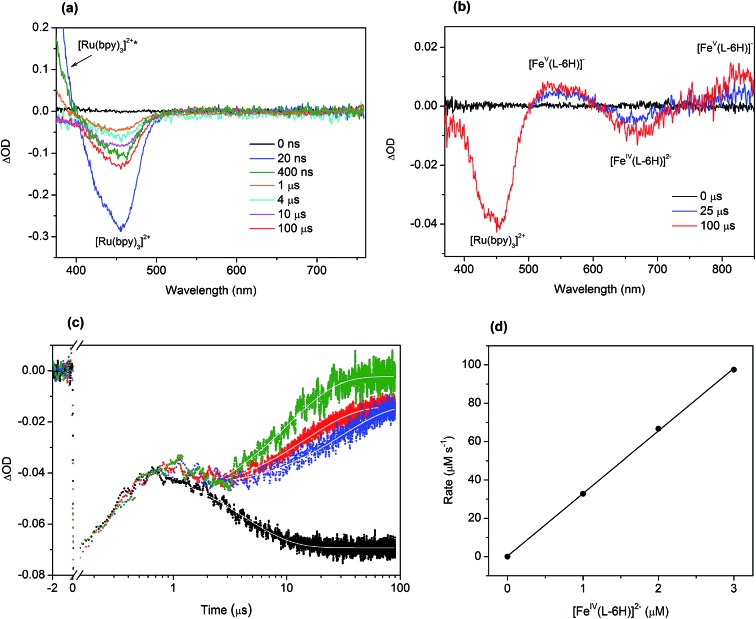
(a) Transient absorption spectra for the solution containing [Ru(bpy)_3_](ClO_4_)_2_ (0.04 mM) and Na_2_S_2_O_8_ (0.4 mM) recorded with different time delays after a pump flash. (b) Transient absorption spectra for the solution containing [Ru(bpy)_3_]^2+^ (0.04 mM), S_2_O_8_^2–^ (0.4 mM) and [Fe^IV^(L–6H)]^2–^ (2 μM) showing the appearance of the bands characteristic of [Fe^V^(L–6H)]^–^. (c) Kinetic traces at 420 nm for the solutions containing [Ru(bpy)_3_](ClO_4_)_2_ (0.04 mM), Na_2_S_2_O_8_ (0.4 mM) and a variable amount of [Fe^IV^(L–6H)]^2–^: black trace – 0 μM, blue trace – 1.0 μM, red trace – 2.0 μM, green trace – 3.0 μM. Fits are shown in white. (d) Dependence of the rate of [Ru(bpy)_3_]^3+^ reduction by [Fe^IV^(L–6H)]^2–^ derived from the kinetic traces at 420 nm on the concentration of the catalyst.

To investigate in detail the light-driven hole transfer from the photo-oxidized ruthenium dye to the iron-based catalyst in aqueous solution, nanosecond transient absorption measurements were performed using the laser pump-probe method. An initial visible-light pump was used to excite [Ru(bpy)_3_]^2+^ (eqn (1)) and start a photochemical reaction, and a probe pulse measured the absorbance of the sample solution after different time delays on nano- and micro-second timescales. For the bare ruthenium dye photo-oxidant system consisting of [Ru(bpy)_3_](ClO_4_)_2_ (0.04 mM) and Na_2_S_2_O_8_ (0.4 mM) without the catalyst, the transient absorption spectra after the pump pulse (460 nm, 30 mJ, 13 ns) resulted in a bleach at 455 nm (Fig. 4a). The intensity of the bleach decreases when the time delay between the pump and probe pulses increases from a few nanoseconds to 1 μs. Moreover, one can observe a shoulder of a new band in the UV region, the intensity of which also decreases on the nanosecond timescale. This transformation can be attributed to the excitation of [Ru(bpy)_3_]^2+^ (*λ*_abs_ = 455 nm) to the triplet state [Ru(bpy)_3_]^2+^* (*λ*_abs_ ∼ 370 nm)^38^ followed by its relaxation. Due to the low oxidation potential of –0.62 V *versus* NHE,^39^ the excited triplet state is oxidatively quenched by S_2_O_8_^2–^ in aqueous solution yielding [Ru(bpy)_3_]^3+^ (eqn (2)). However, we cannot observe generation of [Ru(bpy)_3_]^3+^ in the transient absorption spectra directly, as the extinction coefficient for the broad absorption band of [Ru(bpy)_3_]^3+^ (*λ*_abs_ = 670 nm) is much lower than that for [Ru(bpy)_3_]^2+^ (*λ*_abs_ = 455 nm) (Fig. 2a). After 1 μs, the intensity of the bleach at 455 nm increases since [Ru(bpy)_3_]^2+^ is oxidized by the generated sulfate radical (SO_4_˙^–^) species (eqn (3)).^40^ Under optimized conditions and in the absence of the iron catalyst, SO_4_˙^–^ quantitatively produces the second equivalent of the oxidized ruthenium species, as ascertained from the transient absorption kinetic traces derived at 420 nm (Fig. 4c, black). The trace reflecting reaction between [Ru(bpy)_3_]^2+^ and SO_4_˙^–^ in the solution can be fitted using exponential decay:
4

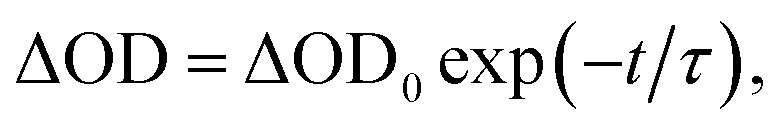

where OD stands for optical density, *t* is the delay between the pump and probe pulses, and *τ* is the lifetime of the transient state, resulting in *τ* = 4 μs. From the OD at 420 nm, the yield of charge-separated products, or cage escape yield, can be calculated using relative actinometry, as previously described.^41^ The cage escape yield for the [Ru(bpy)_3_]^3+^/SO_4_˙^–^ pair is estimated to be 0.75, *i.e.* 75% of the total initial photoinduced electron transfer products are available for subsequent reaction.

The addition of the catalyst [Fe^IV^(L–6H)]^2–^ to the flash-quench mixture followed by excitation with 460 nm laser pulses results in two bleaches at 455 nm and 650 nm and two bands at 550 nm and ∼830 nm observed on the microsecond timescale (Fig. 4b). When comparing these spectral features with the steady-state UV-vis spectra of the catalyst shown in the Fig. 2b and reported previously,^35^,^36^ two positive transient absorbance signals are consistent with the formation of [Fe^V^(L–6H)]^–^, while the negative ΔOD signal at 650 nm corresponds to [Fe^IV^(L–6H)]^2–^:

5 [Fe^IV^(L–6H)]^2–^ + [Ru(bpy)_3_]^3+^ → [Fe^V^(L–6H)]^–^ + [Ru(bpy)_3_]^2+^

The transient absorption kinetic traces derived at 420 nm for the aqueous solutions containing [Ru(bpy)_3_](ClO_4_)_2_ (0.04 mM), Na_2_S_2_O_8_ (0.4 mM) and Na_2_[Fe^IV^(L–6H)] (1.0–3.0 μM) are presented in Fig. 4c. The initial growth of the negative ΔOD signal and its following decrease observed between 10 ns and 1 μs reflect the excitation and relaxation of the photosensitizer, respectively, and occur independently of the presence/concentration of the catalyst. The subsequent decrease in the negative ΔOD on the microsecond timescale can be plausibly fitted with a sum of two exponential functions with parameters *τ*_1_ and *τ*_2_, where *τ*_1_ is fixed to 4 μs (derived from eqn (4)). From the fit of the kinetic traces, the pseudo-first order rate constant *k* = 3.2(1) × 10^10^ s^–1^ of the reaction between the reduced photosensitizer and the catalyst (eqn (5)) has been extracted (Fig. 4d).

Since the triplet excited state of the photosensitizer, [Ru(bpy)_3_]^2+^*, is a very reactive species, one can assume its direct oxidative or reductive quenching by the catalyst, circumventing the persulfate reaction. To investigate this possible scenario, we performed transient emission spectroscopy of the aqueous solution containing [Ru(bpy)_3_](ClO_4_)_2_ (0.04 mM) and Na_2_S_2_O_8_ (0.4 mM). The photosensitizer was selectively excited by the pump pulse (460 nm), and the luminescence trace at *λ*_em_ = 650 nm was recorded (Fig. S4 in the ESI†). When the catalyst Na_2_[Fe^IV^(L–6H)] (2 μM) was added to this mixture, a similar emission trace was obtained. Both traces can be nicely fitted with an exponential decay (eqn (4)) giving lifetimes for the triplet state [Ru(bpy)_3_]^2+^* of 275 ns and 236 ns, respectively. The observed difference suggests that the direct quenching of [Ru(bpy)_3_]^2+^* by [Fe^IV^(L–6H)]^2–^ is possible but as a minor side process at the given concentrations of catalyst, photosensitizer and persulfate. This phenomenon needs to be investigated in future work.

## Discussion

The clathrochelate complex [Fe^IV^(L–6H)]^2–^, which is indefinitely stable in aqueous solutions at pH 1.0–13.0, acts as a homogeneous catalyst for photocatalytic water oxidation by persulfate with [Ru(bpy)_3_]^2+^ as photosensitizer, affording a high TON = 365.^36^ In both chemical-driven and light-driven water oxidation, most iron complexes are known to decompose easily into catalytically active iron oxide nanoparticles.^37^,^42^ However, the use of strong acidic media,^27^–^29^ or modification of the working system may prevent formation of FeO_*x*_ nanoparticles. For example, surface anchoring,^43^ the use of robust polydentate ligands,^33^,^34^ or isolation of a molecular catalyst in a cage of the metal–organic framework (MOF)^44^ has been shown to stabilize the molecular structure of transition metal-based catalysts under oxidative conditions. In our work, the iron ion is encapsulated in a clathrochelate cage that allows sustained water oxidation at nearly neutral pH, in both chemical and photochemical assays. To the best of our knowledge, the complex [Fe^IV^(L–6H)]^2–^ exhibits relatively high catalytic efficiency compared to other molecular iron-based WOCs reported to date (Table 1).

**Table 1:** Comparison of the catalytic performance for selected iron compounds for homogeneous chemical and photochemical water oxidation

Catalyst	Oxidant	pH	TON	TOF (s^–1^)	Ref.
Fe-TAML^*a*^	CAN	1.0	18	1.3	25
Fe-TAML^*a*^	CAN	1.0	93	—	27
Fe-TAML^*a*^	NaIO_4_	1.0	44	—	27
Fe-TAML^*a*^	NaIO_4_	7.0	3	—	27
Fe-TAML^*a*^	CAN	1.0	17	0.03	33
Fe-TAML^*a*^	Ru + S_2_O_8_^2–^ + *hν*^*b*^	8.5	220	0.76	33
[Fe(Pytacn)(OTf)_2_]^*c*^	CAN	0.7	180	0.2	29
[Fe(Mcp)(OTf)_2_]^*d*^	CAN	0.8	360	0.28	28
[Fe(Py5OH)Cl]^–^^*e*^	CAN	1.5	5	0.53	34
[Fe(Py5OH)Cl]^–^^*e*^	[Ru(bpy)_3_]^3+^	8.0	26.5	2.2	34
[Fe(Py5OH)Cl]^–^^*e*^	Ru + S_2_O_8_^2–^ + *hν*^*b*^	8.0	43.5	0.6	34
[Fe(Py5OH)(MeCN)]^2–^^*e*^	CAN	1.5	16	0.75	34
[Fe(Py5OH)(MeCN)]^2–^^*e*^	[Ru(bpy)_3_]^3+^	8.0	7	0.9	34
[Fe(Py5OH)(MeCN)]^2–^^*e*^	Ru + S_2_O_8_^2–^ + *hν*^*b*^	8.0	20	0.6	34
[Fe_2_(Hbb)(OMe)(OAc)]^+^^*f*^	[Ru(bpy)_3_]^3+^	7.2	4	0.012	26
[Fe^IV^(L–6H)]^2–^	[Ru(bpy)_3_]^3+^	8.0	45	3.3	This work, 36
[Fe^IV^(L–6H)]^2–^	Ru + S_2_O_8_^2–^ + *hν*^*b*^	8.0	365	2.27	This work, 36
FeCl_3_^*g*^	Ru + S_2_O_8_^2–^ + *hν*^*b*^	8.0	63	0.6	This work

^*a*^Different tetraamido macrocyclic ligand (TAML) complexes were reported (see original publications for details).

^*b*^Photochemical water oxidation using [Ru(bpy)_3_]^2+^ as photosensitizer and S_2_O_8_^2–^ as sacrificial electron acceptor.

^*c*^Pytacn = 1-(2′-pyridylmethyl)-4,7-dimethyl-1,4,7-triazacyclononane; OTf = triflate anion.

^*d*^Mcp = *N*,*N*′-dimethyl-*N*,*N*′-bis(2-pyridylmethyl)-1,2-cis-diaminocyclohexane; OTf = triflate anion.

^*e*^Py5OH = pyridine-2,6-diylbis(di(pyridin-2-yl)methanol).

^*f*^Hbb = 2,2′-(2-hydroxy-5-methyl-1,3-phenylene)bis(1*H*-benzo[*d*]imidazole-4-carboxylic acid).

^*g*^FeCl_3_ was used as a precatalyst giving iron oxide nanoparticles.

Our transient absorption measurements corroborate the generally accepted scheme of photochemical water oxidation.^12^,^40^ Thus, the excited triplet state of the photosensitizer [Ru(bpy)_3_]^2+^* is quenched by persulfate *via* irreversible electron transfer yielding [Ru(bpy)_3_]^3+^ species. The radical anion SO_4_˙^–^ generated after the quenching reacts with another, non-excited [Ru(bpy)_3_]^2+^ molecule yielding a second equivalent of [Ru(bpy)_3_]^3+^. The oxidized photosensitizer injects its electron vacancy to the catalyst molecule restoring [Ru(bpy)_3_]^2+^, which can give rise to the next cycle of photosensitization and quenching. In the simplified mechanistic model, the hole scavenging reaction must be repeated four times in a row, as the active state of the catalyst must be four-times oxidized to evolve dioxygen from two water molecules. It is usually proposed that a water molecule or hydroxide anion adds to the metal center followed by one- or multi-electron oxidation, addition of a second water molecule and then further oxidation.^29^,^33^ In addition, some deviations from the standard scheme are possible, for example direct oxidation of the catalyst by SO_4_˙^–^. In any case, according to eqn (1)–(3) and (5), two holes are transferred to the catalyst per one absorbed photon; and two photons are needed to drive four-electron oxidation of water to dioxygen in the system, where persulfate is used as the electron acceptor.

One of the factors determining the overall efficiency of the water oxidation system is the rate of the hole transfer reaction shown in eqn (5).^45^ The oxidized form of the photosensitizer [Ru(bpy)_3_]^3+^ is relatively stable in water, but undergoes irreversible decomposition in the presence of SO_4_˙^–^ or other reactive species produced during photocatalysis.^40^,^45^,^46^ Thus, the primary hole scavenging competes with the degradation of [Ru(bpy)_3_]^3+^. In certain systems, the loss of photocatalytic activity has been attributed to the decomposition of the photosensitizer.^47^ Despite the presence of excess amounts of S_2_O_8_^2–^, the catalysis usually lasts for a few minutes and stops long before the sacrificial electron acceptor is fully consumed owing to decomposition of the light absorber. In the case of the system using [Fe^IV^(L–6H)]^2–^, the catalyst reduces [Ru(bpy)_3_]^3+^ relatively quickly compared to other systems.^40^,^48^,^49^ Though there are a limited number of reported studies devoted to investigation of the hole scavenging activity of molecular WOCs published to-date, the reported TOF is comparatively high (Table 1). We have also found that addition of a fresh portion of [Ru(bpy)_3_]^2+^ to the photocatalytic mixture after >350 turnovers does not reactivate oxygen evolution, indicating that it is not the light absorber, but rather the catalyst, that degrades in the course of water oxidation.

A key challenge for us is to decipher the mechanism of water oxidation catalyzed by the clathrochelate complex [Fe^IV^(L–6H)]^2–^. Our kinetic studies suggest that one molecule of catalyst is involved in oxygen evolution (Fig. 3b). Thus, the typically rate-determining O–O bond formation takes place intramolecularly. According to the generally accepted mechanistic pathway for water oxidation by molecular iron based catalysts, oxidation of an Fe-aqua complex leads to the formation of an oxo-complex Fe=O; then, nucleophilic attack of a second water molecule at the oxo O atom gives rise to the O–O bond.^50^ This mechanism is feasible for complexes of Fe, the coordination spheres of which are not saturated (*e.g.*, square planar Fe-TAML complexes). For [Fe(Py5OH)Cl]^–^ featuring a hexacoordinated metal ion, it was proposed that the binding mode of the ligand changes during catalysis so that a vacant coordination site opens to form an Fe-aqua complex.^34^ However, such a mechanism would not be relevant to [Fe^IV^(L–6H)]^2–^ with the robust clathrochelate ligand. On the other hand, one might assume a heptacoordinated Fe-aqua intermediate, as was found for Ru-based molecular WOCs.^51^ Additionally, we could not completely exclude the outer-sphere electron transfer mechanism, as has been proposed for the related cobalt(ii) clathrochelates, which catalyzed water reduction to hydrogen in the [Ru(bpy)_3_]^2+^ photo-reductant system.^52^ Finally, one could consider an alternative pathway involving α,β-dicarbonyl OH adducts, which could react further to generate intermediate dioxetanes or endoperoxides. This implies that O–O bond formation occurs on the ligand, whereas the iron ion acts as an electron shuttle (since Fe in [Fe(L–6H)]^*n*–^ may possess oxidation states from +3 till +5, and apparently even till +6 as can be seen in electrochemical experiments).^35^ This kind of mechanism was first suggested for the ruthenium “blue dimer”, where the O–O bond was proposed to be formed on carbon as a fragment of the four-membered endoperoxide ring C_2_O_2_.^53^ Though we could characterize one of the possible intermediates [Fe^V^(L–6H)]^–^, the mechanism of water oxidation using the clathrochelate catalyst remains elusive. Intermediate species beyond Fe^V^ seem to be very reactive, and hence have so far not been observable experimentally. To help to understand the catalytic mechanism, future work will include changes to the ligand structure in combination with computational studies.

## Summary

The visible light-driven hole transfer from tris(bipyridine)ruthenium dye to the catalyst [Fe^IV^(L–6H)]^2–^ in an aqueous solution occurs with a pseudo-first-order rate constant 3.2(1) × 10^10^ s^–1^. To our knowledge, this is the most efficient hole scavenging reported for molecular WOCs in the system utilizing [Ru(bpy)_3_]^2+^ as the light absorber and S_2_O_8_^2–^ as the electron acceptor at nearly neutral pH. Due to the fast hole injection, the oxidized photosensitizer does not accumulate, allowing it to avoid oxidative decomposition. This finding is in harmony with the record high TON reached in photochemical water oxidation by using [Fe^IV^(L–6H)]^2–^.^36^ The efficiency of this catalyst might be further improved by introducing bulky substituents at the methylene groups of the ligand to achieve higher stability. Together with the mechanistic studies, this work is currently ongoing.

## Conflicts of interest

There are no conflicts of interest to declare.

## Supplementary Material

Supplementary information
