# Supplementary material for: Photoinduced hole transfer from tris(bipyridine)ruthenium dye to a high-valent iron-based water oxidation catalyst
Source: Faraday Discuss. 2019 Apr 5;215:162–74. doi: 10.1039/c8fd00167g (PMC6677028; doi:10.1039/c8fd00167g)
Supplement: Supplementary file 1 [file FD-215-C8FD00167G-s001.pdf]

Electronic Supplementary Material (ESI) for Faraday Discussions.
This journal is © The Royal Society of Chemistry 2018

Electronic Supplementary Information

Photoinduced hole transfer from tris(bipyridine)ruthenium dye to a high-valent iron-based water oxidation catalyst

Sergii I. Shylin,*[a] Mariia V. Pavliuk,[a] Luca D'Amario,[a,b] Igor O. Fritsky*[c,d] and Gustav Berggren*[a]

[a] Department of Chemistry – Ångström Laboratory, Uppsala University, P.O. Box 523, 75120 Uppsala, Sweden. E-mail: sergii.shylin@kemi.uu.se; gustav.berggren@kemi.uu.se
[b] Physics Department, Free University Berlin, Arnimallee 14, 14195 Berlin, Germany
[c] Department of Chemistry, Taras Shevchenko National University of Kyiv, Volodymyrska 64, 01601 Kiev, Ukraine. E-mail: ifritsky@univ.kiev.ua
[d] PBMR Labs Ukraine, Murmanska 1, 02094 Kiev, Ukraine

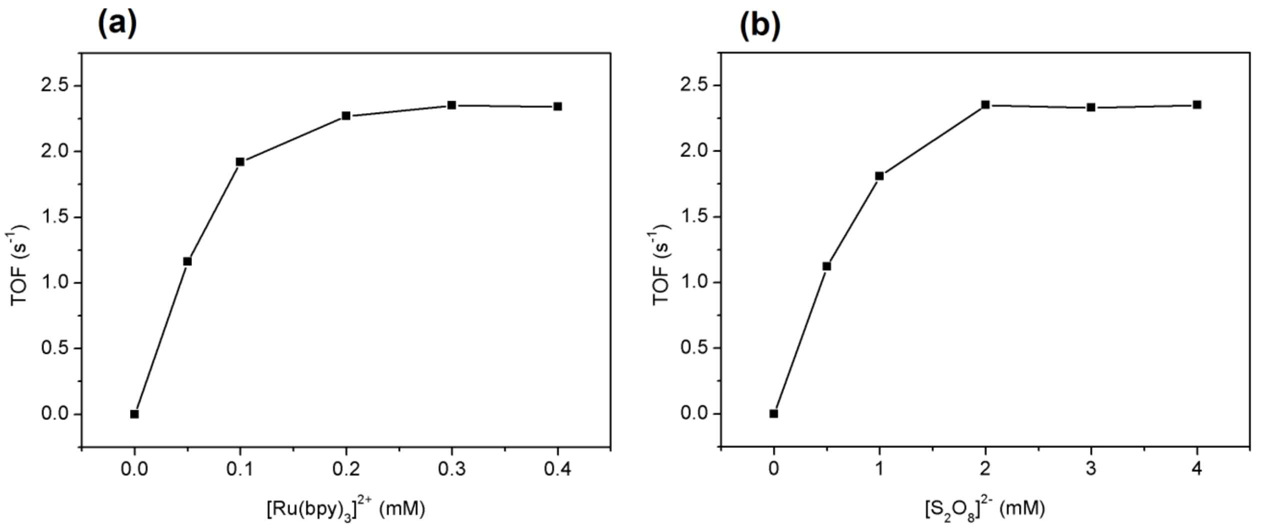

Figure S1. (a) Turnover frequency (TOF) dependence on the concentration of the photosensitizer for photochemical water oxidation. Concentrations of $[Fe^{IV}(L–6H)]^{2–}$ and $S_2O_8^{2–}$ were 1 μM and 2 mM respectively. (b) TOF dependence on the concentration of the sacrificial electron donor. Concentrations of $[Fe^{IV}(L–6H)]^{2–}$ and $[Ru(bpy)_3]^{2+}$ were 1 μM and 0.3 mM. All experiments were done in borate buffer (0.1 M, pH 8.0).

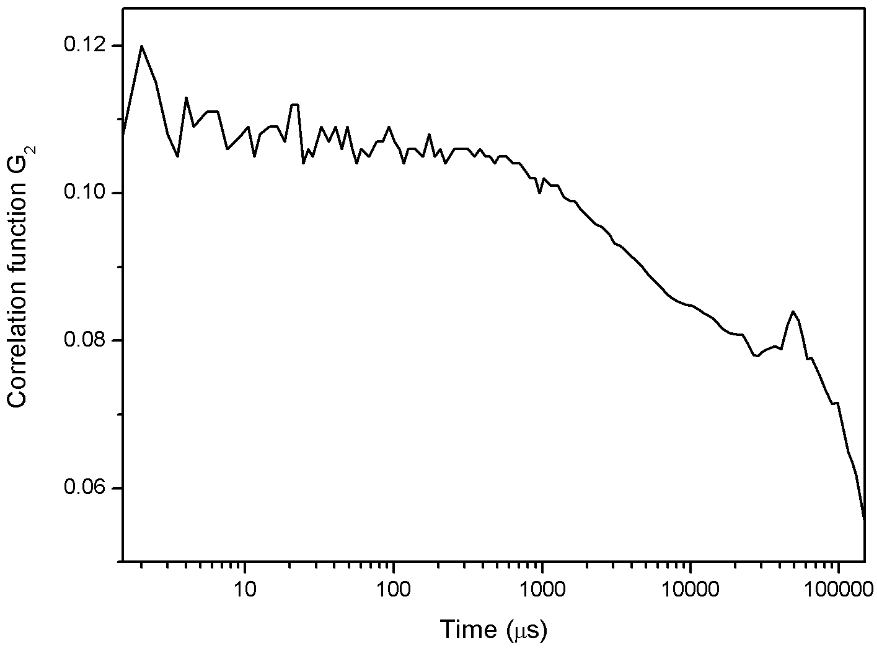

Figure S2. Dynamic light scattering (DLS) correlation function $G_2(\tau)$ for the solution containing $[Ru(bpy)_3]^{2+}$ (0.2 mM), $S_2O_8^{2–}$ (2 mM), and $[Fe^{IV}(L–6H)]^{2–}$ (0.01 mM) in borate buffer (pH 8.0) recorded after >300 turnovers. No particles are observed since $G_2(0) \sim 0.11$ (for heterogeneous systems, typical $G_2(0)$ is 0.6÷1.0). The size measurement range was 0.3 nm – 10 μm.

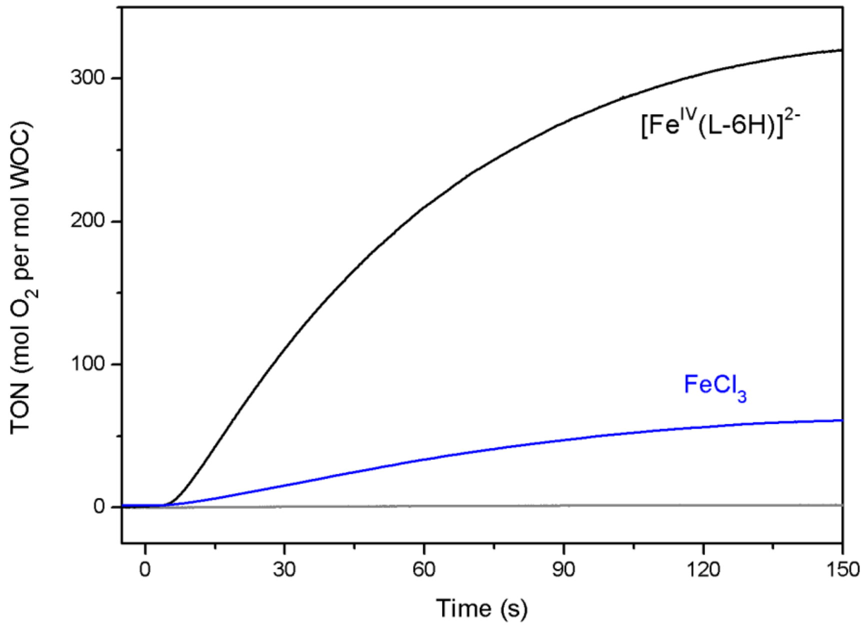

Figure S3. Traces of oxygen evolution for [Fe^{IV}(L–6H)]$^{2-}$ (1 μM) and $FeCl_3$ (1 μM). The latter was used as a precatalyst giving catalytically active hematite nanoparticles at pH 8.0. Concentrations of $[Ru(bpy)_3]^{2+}$ and $S_2O_8^{2-}$ were 0.2 mM and 2 mM respectively. The background oxygen trace is shown in grey. All experiments were done in borate buffer (0.1 M, pH 8.0).

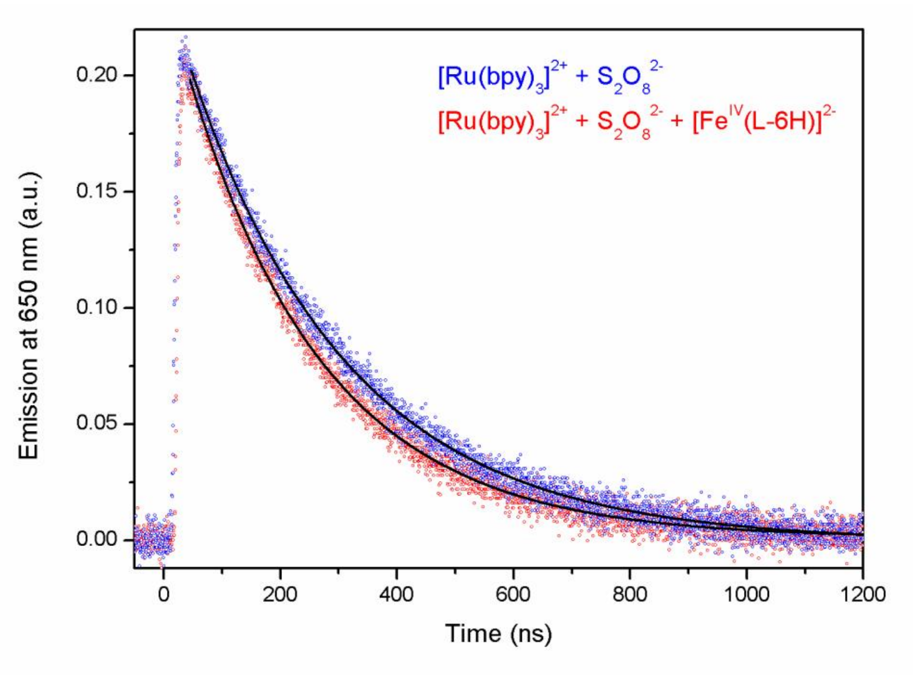

Figure S4. Kinetic traces of $[Ru(bpy)_3]^{2+}$ luminescence at 650 nm for solutions containing $[Ru(bpy)_3](ClO_4)_2$ (0.04 mM), $Na_2S_2O_8$ (0.4 mM) with and without the catalyst [Fe^{IV}(L–6H)]$^{2-}$ (2 μM). Fits are shown in black.

Cage escape yield

The efficiency of charge separation, or cage escape yield, was estimated based on the following mechanistic model:

$$[Ru(bpy)_3]^{2+} + h\nu \rightarrow [Ru(bpy)_3]^{2+*} \qquad (S1)$$

$$[Ru(bpy)_3]^{2+*} + S_2O_8^{2-} \rightarrow \{[Ru(bpy)_3]^{2+}\cdots S_2O_8^{2-}\}^* \rightarrow [Ru(bpy)_3]^{3+} + SO_4^{2-} + SO_4^{\bullet-} \qquad (S2)$$

$$\{[Ru(bpy)_3]^{2+}\cdots S_2O_8^{2-}\}^* \rightarrow [Ru(bpy)_3]^{2+} + S_2O_8^{2-} \qquad (S3)$$

$$[Ru(bpy)_3]^{2+} + SO_4^{\bullet-} \rightarrow [Ru(bpy)_3]^{3+} + SO_4^{2-} \qquad (S4)$$

It is proposed[1] that the excited triplet state of $[Ru(bpy)_3]^{2+*}$ and $S_2O_8^{2-}$ form a complex ("cage") that is followed by O–O bond cleavage yielding $[Ru(bpy)_3]^{3+}$, SO_4^{2-} and $SO_4^{\bullet-}$ (eq. S2). The generated sulfate radical is available for generation of the second equivalent of $[Ru(bpy)_3]^{3+}$. However, the complex may also dissociate into $[Ru(bpy)_3]^{2+}$ and $S_2O_8^{2-}$, thus without forming charge separated products (eq. S3). Within this model, the cage escape yield can be calculated as amount of $[Ru(bpy)_3]^{2+}$ reacting with $SO_4^{\bullet-}$ (eq. S4) relative to amount of $[Ru(bpy)_3]^{2+}$ giving the complex $\{[Ru(bpy)_3]^{2+}\cdots S_2O_8^{2-}\}^*$:

$$\eta = \frac{\Delta OD_{\mu s} - \Delta OD_{ns}}{\Delta OD_{ns}} = \frac{-0.07+0.04}{-0.04} = 0.75,$$

where ΔOD_{ns} and $\Delta OD_{\mu s}$ stand for transient optical density derived at 420 nm (absorption of $[Ru(bpy)_3]^{2+}$) at 700 ns and 80 μs respectively (Figure 4c in the main text, black trace).

[1] A. L. Kaledin, Z. Huang, Y. V Geletii, T. Lian, C. L. Hill and D. G. Musaev, *J. Phys. Chem. A*, 2010, **114**, 73-80.
